# Supplementary figures and images for: Multi-omics signatures of alcohol use disorder in the dorsal and ventral striatum
Source: Transl Psychiatry. 2022 May 6;12:190. doi: 10.1038/s41398-022-01959-1 (PMC9076849; doi:10.1038/s41398-022-01959-1)

A) Caudate Nucleus

$$\lambda = 1.78$$

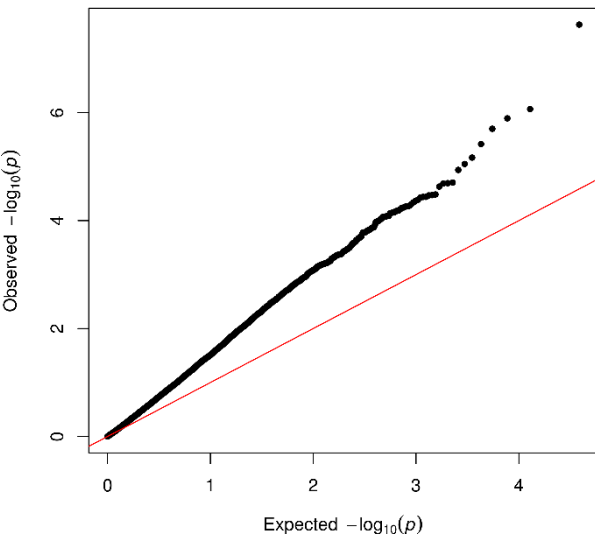

B) Putamen

$$\lambda = 1.63$$

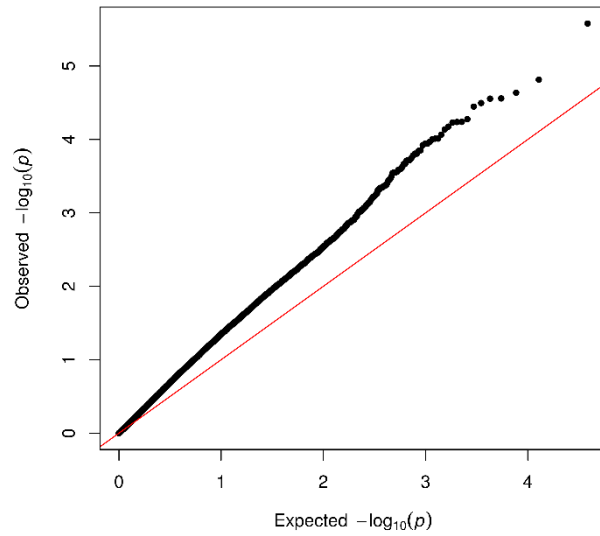

C) Ventral Striatum

$$\lambda = 1.22$$

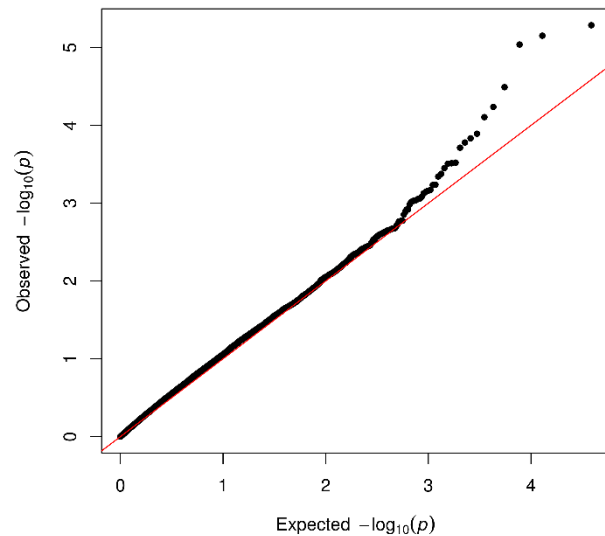

Supplement: Supplementary file 2 — Supplementary Figure 1 [file 41398_2022_1959_MOESM2_ESM.pdf]

A) Caudate Nucleus

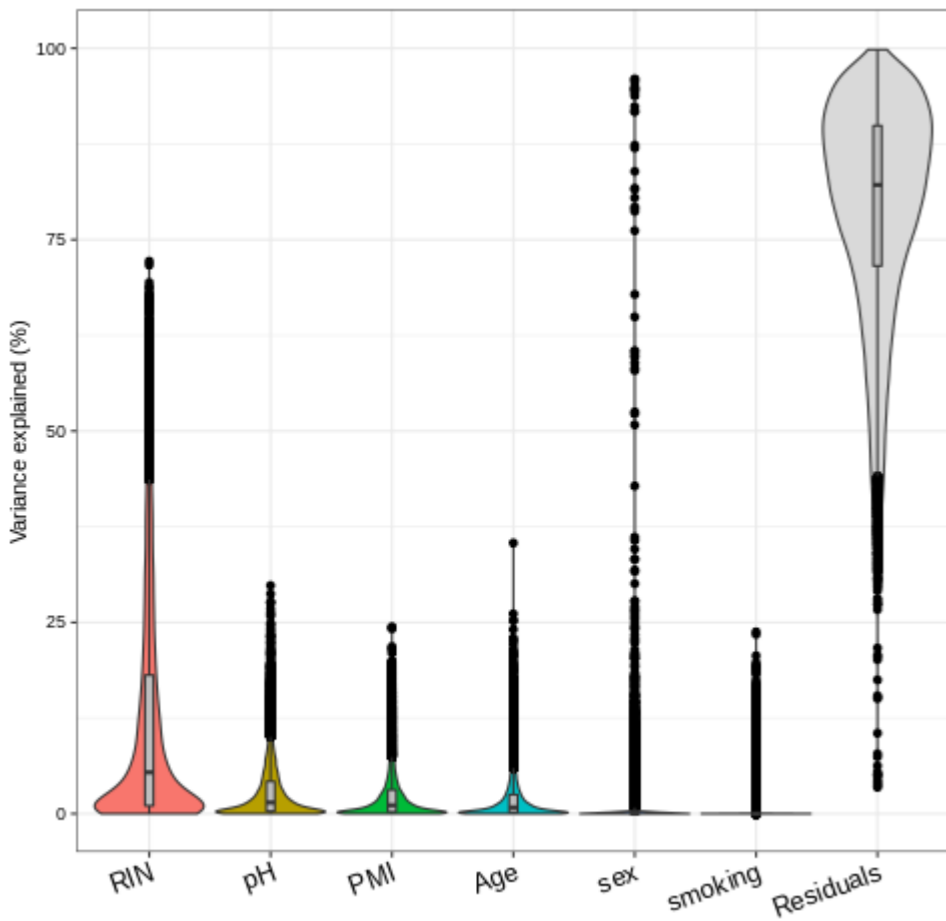

B) Putamen

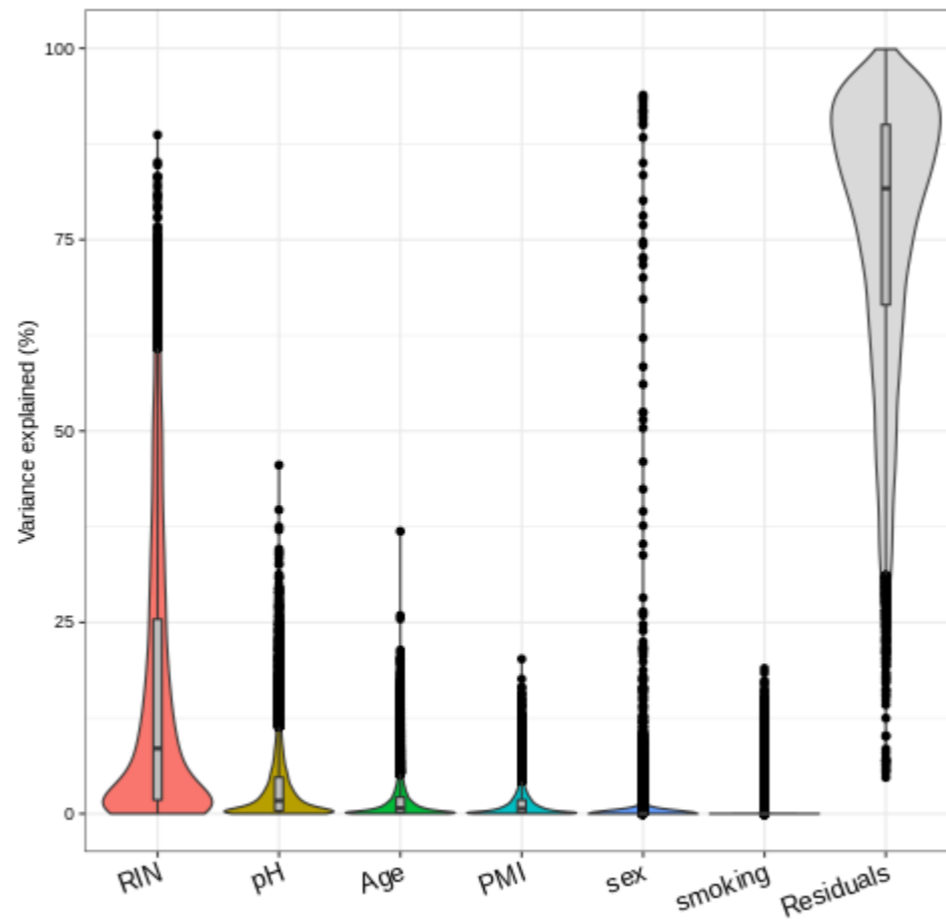

C) Ventral Striatum

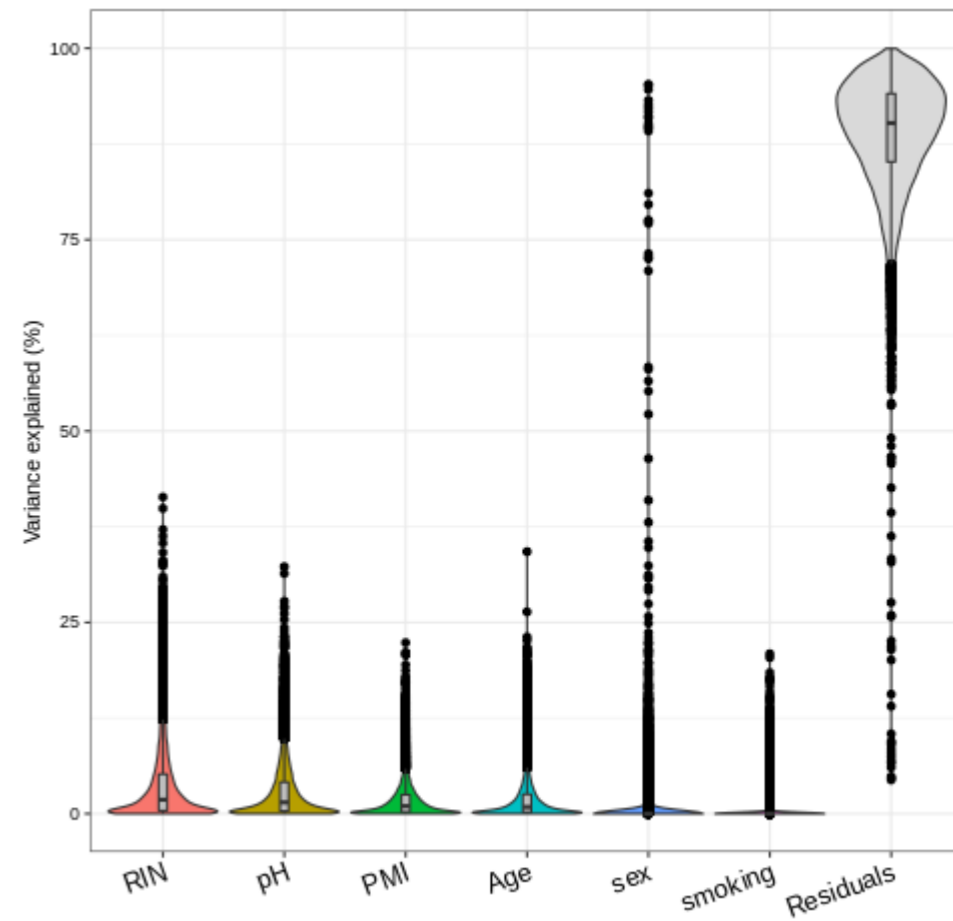

Supplement: Supplementary file 3 — Supplementary Figure 2 [file 41398_2022_1959_MOESM3_ESM.pdf]

A) Caudate Nucleus

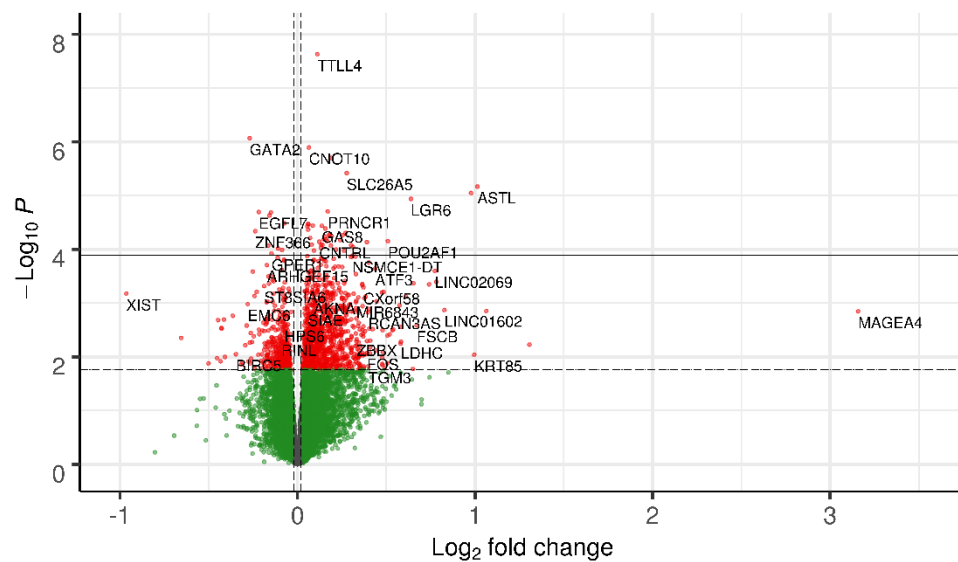

B) Putamen

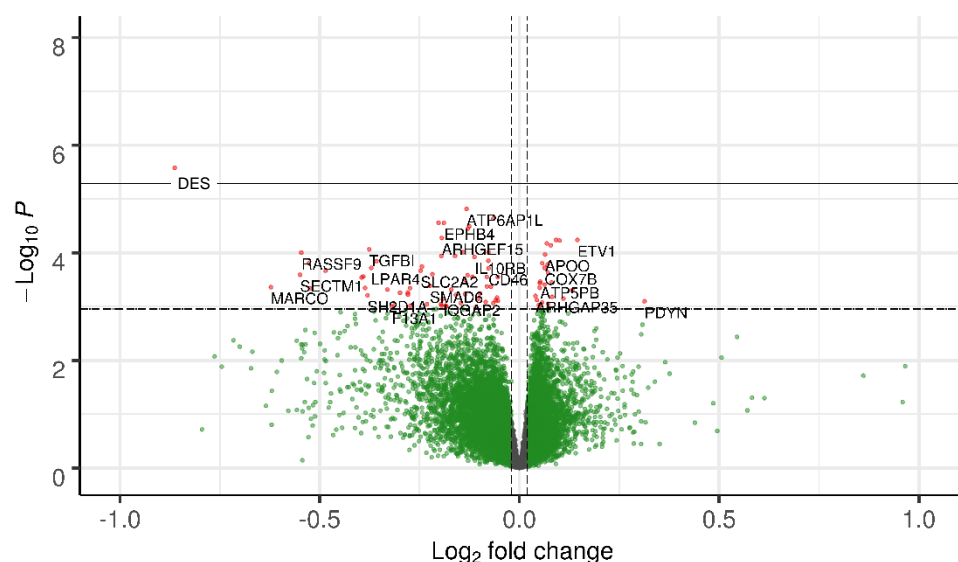

C) Ventral Striatum

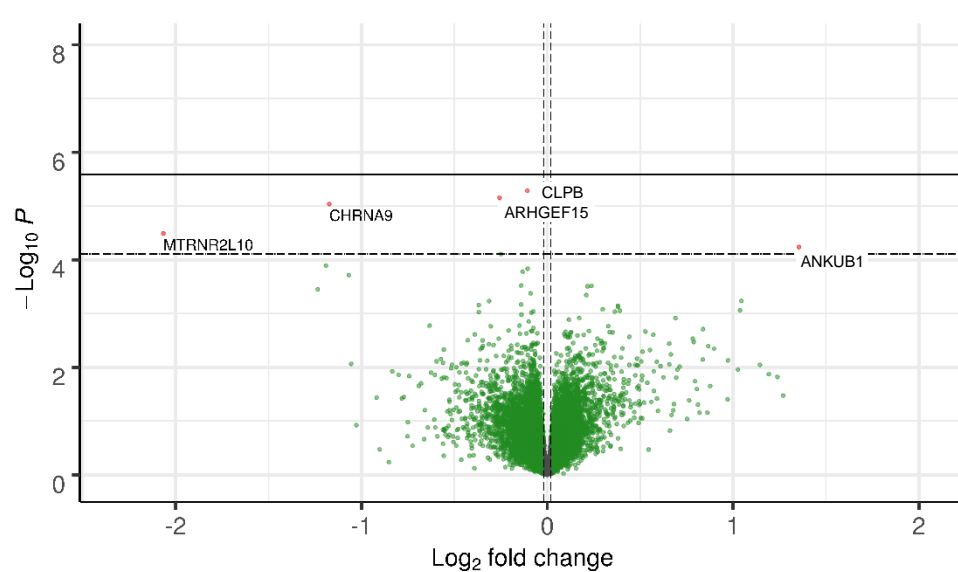

Supplement: Supplementary file 4 — Supplementary Figure 3 [file 41398_2022_1959_MOESM4_ESM.pdf]

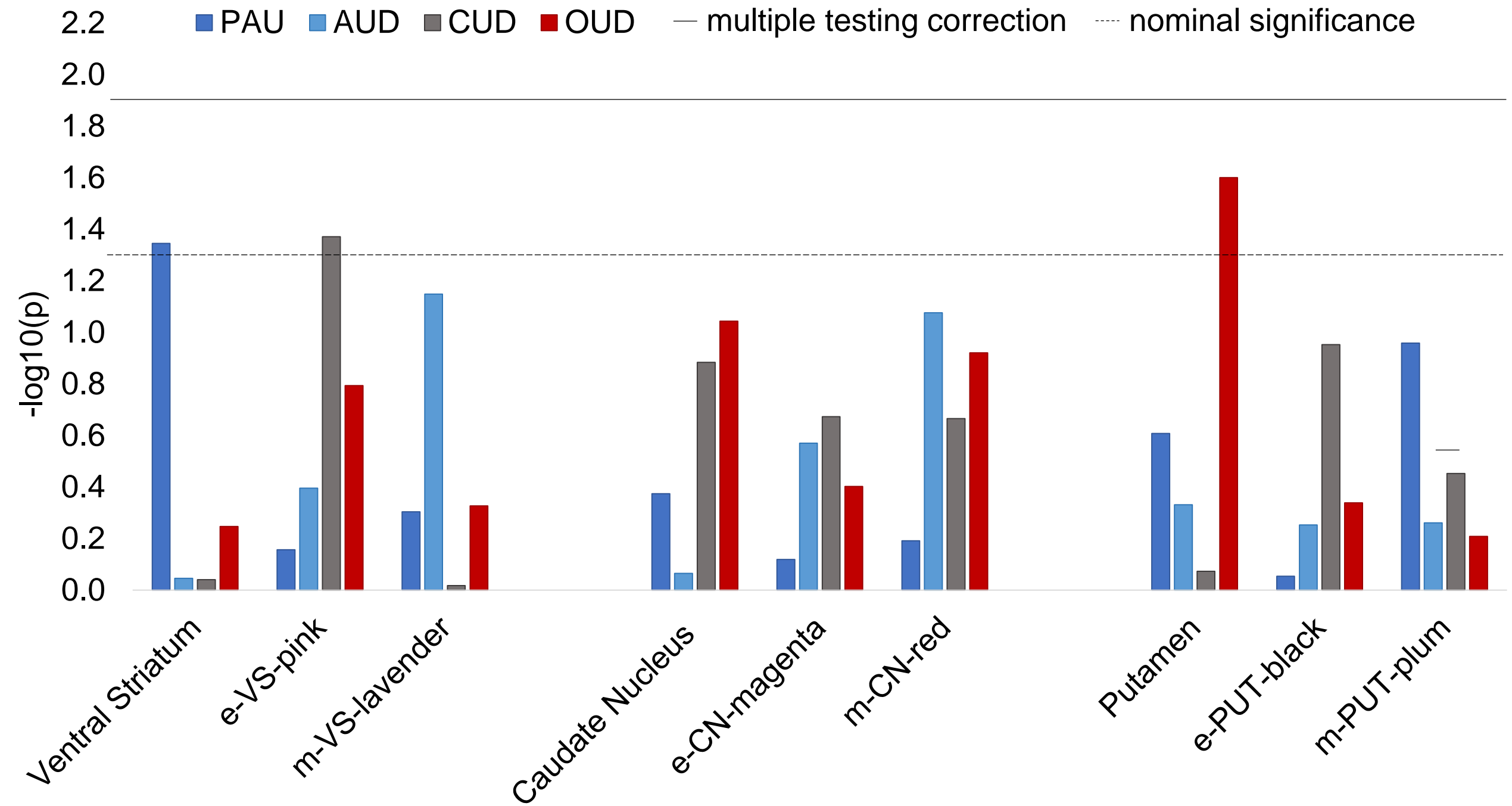

Supplement: Supplementary file 7 — Supplementary Figure 6 [file 41398_2022_1959_MOESM7_ESM.pdf]
