# Supplementary material for: Multi-omics signatures of alcohol use disorder in the dorsal and ventral striatum
Source: Transl Psychiatry. 2022 May 6;12:190. doi: 10.1038/s41398-022-01959-1 (PMC9076849; doi:10.1038/s41398-022-01959-1)
Supplement: Supplementary file 5 — Supplementary Figure 4 [file 41398_2022_1959_MOESM5_ESM.pdf]

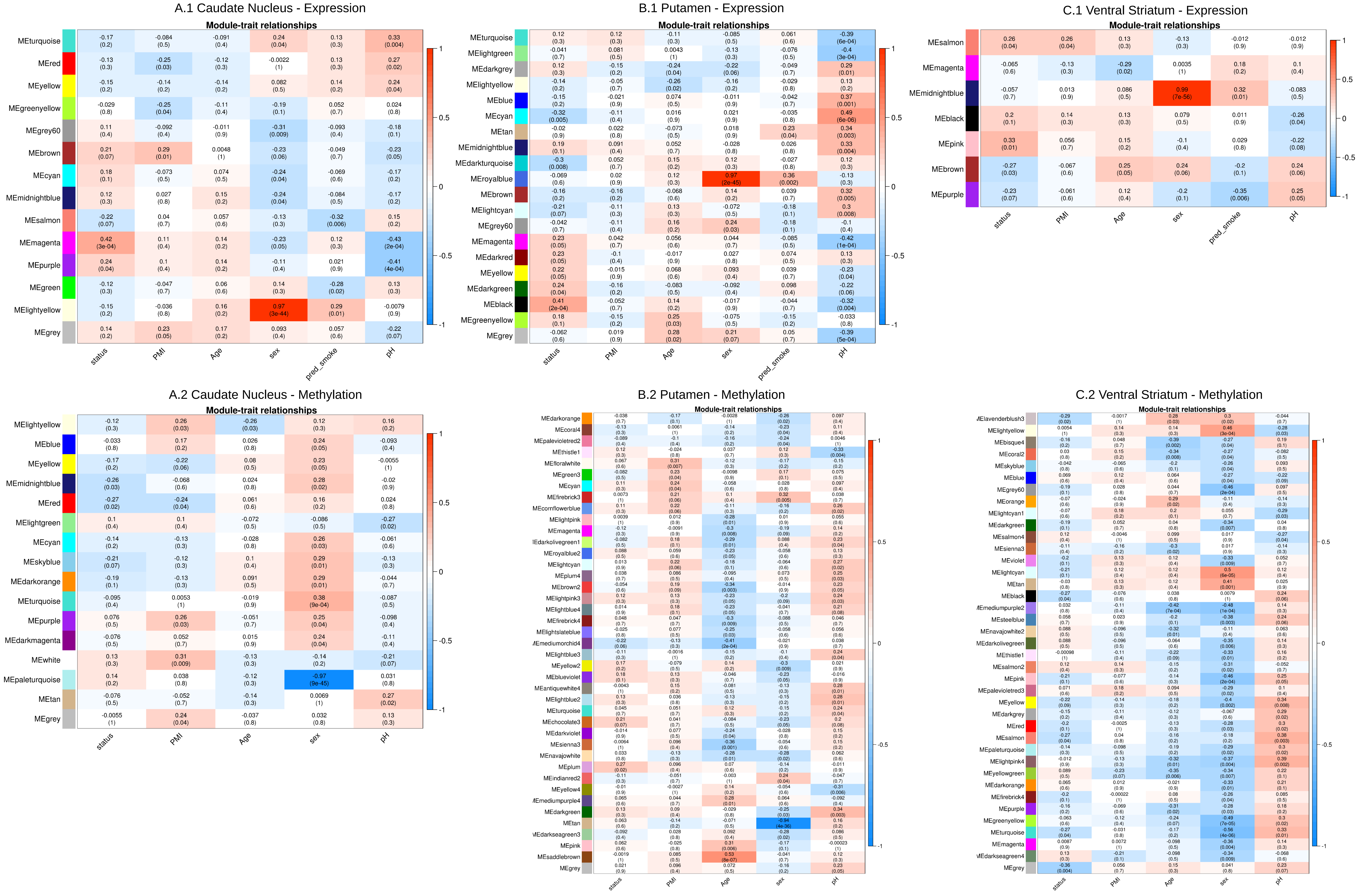
A.1 Caudate Nucleus - Expression
Module-trait relationships
MEturquoise
MEred
MEyellow
MEgreenyellow
MEgrey60
MEbrown
MEcyan
MEmidnightblue
MEsalmon
MEmagenta
MEpurple
MEgreen
MElightyellow
MEgrey
status
PMI
Age
sex
pred_smoke
pH
B.1 Putamen - Expression
Module-trait relationships
MEturquoise
MElightgreen
MEdarkgrey
MElightyellow
MEblue
MEcyan
MEtan
MEmidnightblue
MEdarkturquoise
MEroyalblue
MEbrown
MElightcyan
MEgrey60
MEmagenta
MEdarkred
MEyellow
MEdarkgreen
MEblack
MEgreenyellow
MEgrey
C.1 Ventral Striatum - Expression
Module-trait relationships
MEsalmon
MEmagenta
MEmidnightblue
MEblack
MEpink
MEbrown
MEpurple
A.2 Caudate Nucleus - Methylation
Module-trait relationships
MElightyellow
MEblue
MEyellow
MEmidnightblue
MEred
MElightgreen
MEcyan
MEskyblue
MEdarkorange
MEturquoise
MEpurple
MEdarkmagenta
MEwhite
MEpaleturquoise
MEtan
MEgrey
B.2 Putamen - Methylation
Module-trait relationships
C.2 Ventral Striatum - Methylation
Module-trait relationships
