# Supplementary material for: Multi-omics signatures of alcohol use disorder in the dorsal and ventral striatum
Source: Transl Psychiatry. 2022 May 6;12:190. doi: 10.1038/s41398-022-01959-1 (PMC9076849; doi:10.1038/s41398-022-01959-1)
Supplement: Supplementary file 6 — Supplementary Figure 5 [file 41398_2022_1959_MOESM6_ESM.pdf]

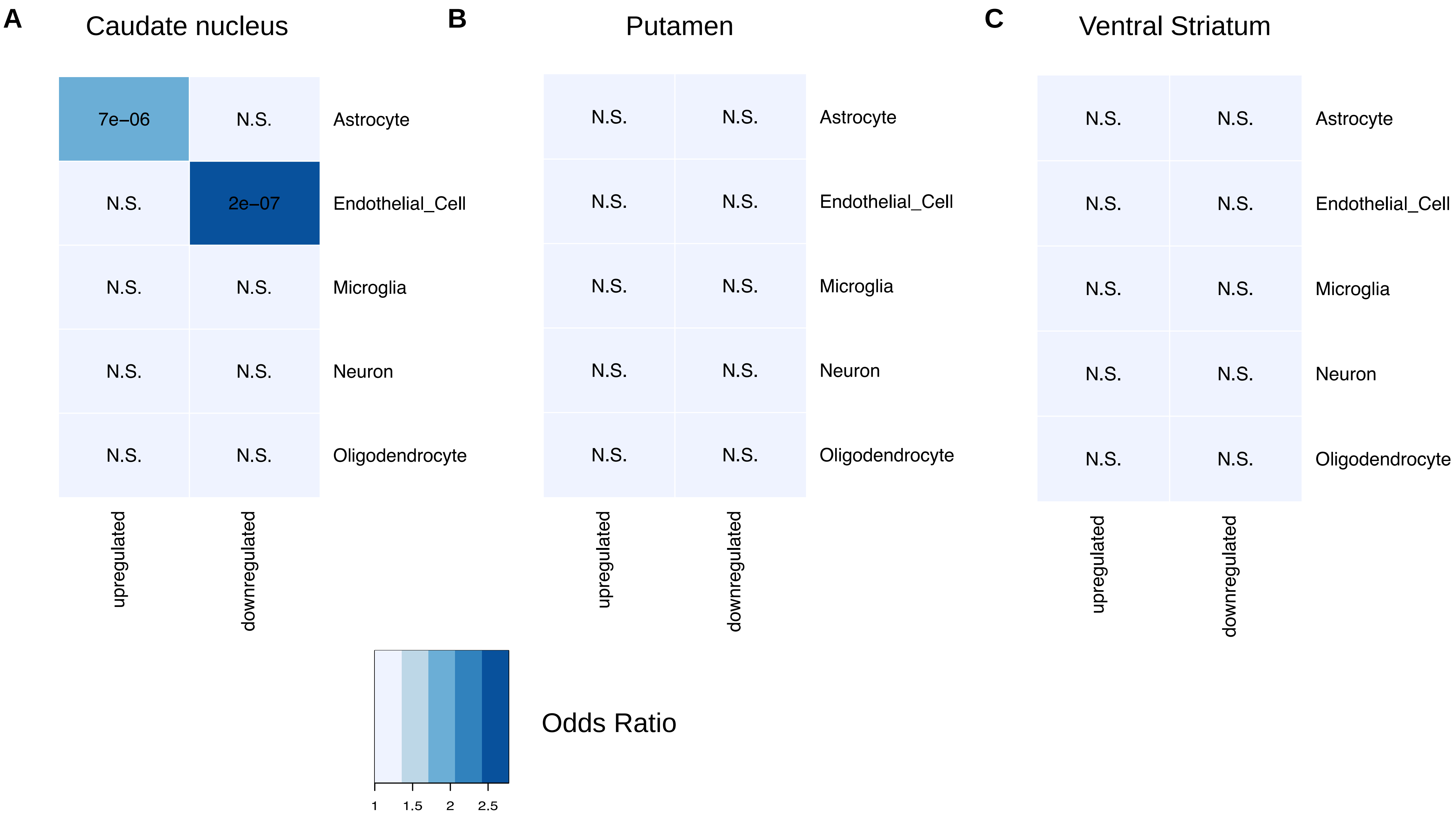
A
Caudate nucleus
7e−06
N.S.
Astrocyte
N.S.
2e−07
Endothelial_Cell
N.S.
N.S.
Microglia
N.S.
N.S.
Neuron
N.S.
N.S.
Oligodendrocyte
upregulated
downregulated
B
Putamen
N.S.
N.S.
Astrocyte
N.S.
N.S.
Endothelial_Cell
N.S.
N.S.
Microglia
N.S.
N.S.
Neuron
N.S.
N.S.
Oligodendrocyte
upregulated
downregulated
C
Ventral Striatum
N.S.
N.S.
Astrocyte
N.S.
N.S.
Endothelial_Cell
N.S.
N.S.
Microglia
N.S.
N.S.
Neuron
N.S.
N.S.
Oligodendrocyte
upregulated
downregulated
Odds Ratio
1
1.5
2
2.5
